# Herb-Drug Interaction between the Traditional Hepatoprotective Formulation and Sorafenib on Hepatotoxicity, Histopathology and Pharmacokinetics in Rats

**DOI:** 10.3390/molecules22071034

**Published:** 2017-06-22

**Authors:** Chin-Tsung Ting, Yung-Yi Cheng, Tung-Hu Tsai

**Affiliations:** 1 Institute of Traditional Medicine, School of Medicine, National Yang-Ming University, Taipei 112, Taiwan; DAB74@tpech.gov.tw (C.-T.T.); vininecheng@gmail.com (Y.-Y.C.); 2 Division of Gastrointestinal Surgery, Department of Surgery, Ren-Ai Branch, Taipei City Hospital, Taipei 10629, Taiwan; 3 Graduate Institute of Acupuncture Science, China Medical University, Taichung 40402, Taiwan; 4 School of Pharmacy, College of Pharmacy, Kaohsiung Medical University, Kaohsiung 80708, Taiwan; 5 Department of Chemical Engineering, National United University, Miaoli 36063, Taiwan

**Keywords:** hepatocellular carcinoma, Chinese herbal medicine, Radix Gentianae formulation, sorafenib, pharmacokinetics

## Abstract

Sorafenib has been used as a standard therapy for advanced hepatocellular carcinoma (HCC). In Asia, patients with HCC are potentially treated with the combination of sorafenib and Chinese herbal medicines to improve the efficiency and reduce the side effects of sorafenib. However, limited information about the herb-drug interactions is available. We hypothesize that the Chinese herbal medicine may exert hepatoprotective effects on the sorafenib-treated group. The aim of this study is to investigate the pharmacokinetic mechanism of drug-drug interactions of sorafenib including interacting with hepatoprotective formulation, Long-Dan-Xie-Gan-Tang formulation (LDXGT) and with two cytochrome P450 3A4 (CYP3A4) inhibitors, grapefruit juice and ketoconazole. Liver enzyme levels and histopathology of liver slices were used to evaluate sorafenib-induced hepatotoxicity and the potential hepatoprotective effects of the LDXGT formulation on subjects treated with the combination of sorafenib and the herbal medicine. In this study, a validated HPLC-photodiode array analytical system was developed for the pharmacokinetic study of sorafenib in rats. As the result of the pharmacokinetic data, pretreatment with the LDXGT formulation did not significantly interact with sorafenib compared with sorafenib oral administration alone. Furthermore, grapefruit juice and ketoconazole did not significantly affect sorafenib metabolism. Furthermore, pretreatment with variable, single or repeat doses of the LDXGT formulation did not suppress or exacerbate the sorafenib-induced hepatotoxicity and histopathological alterations. According to these results, the LDXGT formulation is safe, but has no beneficial effects on sorafenib-induced hepatotoxicity. A detailed clinical trial should be performed to further evaluate the efficacy or adverse effects of the LDXGT formulation in combination with sorafenib in humans.

## 1. Introduction

Hepatocellular carcinoma (HCC) is the third most common cause of cancer-related death, and its global annual incidence is rising [1,2]. Hepatitis B virus (HBV) and hepatitis C virus (HCV) infections are recognized as the major risk factors for the development of HCC [2]. The prognosis and outcome after treatment for HCC are generally related to the tumor stage at presentation. To date, the best therapy for HCC is still surgical hepatic resection [3,4]. Other treatments, such as percutaneous ablation [5] and liver transplantation [6], offer a high probability of complete response in patients with early and intermediate stage HCC. Recent progress in diagnostic tools and surgical techniques has resulted in considerably improved morbidity and mortality rates. However, less than 30% of patients with HCC can be treated with curative therapy, and the overall five-year survival rate following resection has remained as low as 35–50% due to the high recurrence rate in the remnant liver. In the majority of patients with HCC who do not meet the criteria for curative therapies, recurrence after curative therapies or extra-hepatic spread should be treated with another palliative treatment, such as transarterial chemoembolization (TACE), chemo-radiotherapy or alternative therapies.

Sorafenib, the only FDA-approved multi-kinase inhibitor, has been used as a standard medical therapy for advanced HCC. It suppresses tumor angiogenesis by blocking vascular endothelial growth factor receptor (VEGFR) and platelet-derived growth factor receptor (PDGFR) signaling. Sorafenib also exerts anti-proliferative effects on HCC cells by inhibiting the receptor tyrosine kinases KIT and FLT-3 and the serine/threonine kinases in the RAF/MEK/ERK pathway [7]. Its efficacy has been proven in two large-scale randomized control studies treating patients with advanced HCC [8,9]. However, the efficacy of sorafenib alone is still far from satisfactory. The identification of drugs that may synergize with sorafenib may help to improve its efficacy.

In both Eastern and Western countries, chronic hepatitis and its clinical sequelae are serious health problems and are closely related to HCC [10,11]. Among subjects with HCC complicated with chronic hepatitis or cirrhosis, viral hepatitis B and C are the major etiologies, and these patients receive antiviral agents and other treatments to control hepatitis [10,11,12]. Meanwhile, many patients (approximately 38–42% of the global population with chronic hepatitis) seek assistance from complementary and alternative medicines, possibly due to their culture, concerns regarding the side effects of interferon and/or anti-viral agents and the belief that complementary and alternative medicines protect the liver [13,14,15,16]. Chinese herbal medicine, an important category of complementary and alternative medicine, has been proven to be an efficacious and safe treatment option for patients with chronic hepatitis in some clinical trials and has been popularly used in Asia and even Western countries [13,14,17,18,19].

According to the 2002 large-scale pharmacoepidemiological study obtained from the National Health Insurance database in Taiwan, Long-Dan-Xie-Gan-Tang (LDXGT) and *Salvia miltiorrhiza* (Chinese herbal name: Dan-Shen) were the most commonly-prescribed Chinese herbal formula and single herbal drug for chronic hepatitis therapy, respectively [20]. The traditional Chinese prescription LDXGT is composed of ten important plants: Radix Gentianae, Radix Scutellariae, Fructus Gardeniae, Rhizoma Alismatis, Caulis Hocquartiae, Semen Plantaginis, Radix Angelicae Sinensis, Radix Rehmanniae, Radix Bupleuri and Radix Glycyrrhizae. LDXGT is a famous Chinese prescription and is widely used in Chinese medicine due to its anti-inflammatory, anti-infection, anti-bacterial, anti-allergy, hepatoprotective, cholagogue and immunostimulant functions [21,22,23,24]. The herbal drugs contained in this formula are reported to have multiple effects, such as anti-hepatitis B virus or other virus (*Scutellaria baicalensis*, *Bupleurum chinense*, *Glycyrrhiza uralensis* and *Gentiana scabra*), anti-inflammatory (*Gardenia jasminoides*) and anti-oxidant or immunomodulatory effects (*Alisma plantago*, *Plantago asiatica*, *Akebia trifoliate*, *Rehmannia glutinosa*, *Angelica sinensis*, *Bupleurum chinense* and *Glycyrrhiza uralensis*) [25,26,27,28,29,30,31,32,33,34]. This formula exerts a hepatoprotective effect on CCl_4_-induced hepatic injury in rats, and the serum aspartate transaminase (AST) and alanine transaminase (ALT) levels are significantly decreased following treatment with the Radix Gentianae formulation [35]. In addition, the combination of the Radix Gentianae formulation and IFN-α significantly improves the negative conversion rates of HBeAg in the treatment of chronic hepatitis B infection [36]. The LDXGT formulation has not only been recorded in ancient Chinese classics of medicine as having inhibitory effects on inflammatory diseases of the liver or gall bladder, but also has been shown to have anti-inflammatory and anti-herpetic virus effects [21,22]. Nevertheless, herbal medicine contains numerous components causing various effects. Some of the herbal ingredients are able to provide the benefit of disease treatments, yet others are harmful if ingested. Several pieces of clinical evidence revealed that a few herbs result in hepatotoxicity, such as Jin Bu Huan, a well-known analgesic herb [37]. With the rising concern about the application of Chinese herbal medicine, the herb-drug interaction and hepatotoxicity are increasingly vital issue. Therefore, clarifying the safety issue of the practice of Chinese herbal medicine by studying the pharmacokinetics, pharmacodynamics and herb-drug interaction is critical.

According to the aforementioned information, there is potential risk of sorafenib co-administrated with the LDXGT formulation in HCC outpatients. Therefore, a well-designed study aimed at evaluating the potential interaction of the LDXGT formulation with sorafenib regarding hepatotoxicity, histopathology and pharmacokinetics must be conducted. In addition, many patients primarily use the formula for hepatitis control before and after they are diagnosed with HCC. The herb-drug interaction between traditional Chinese herbal medicines and Western medicines is still unknown. We hypothesize that the herbal medicine may exert hepatoprotective effects on the sorafenib-treated group. The aim of this study is to investigate the pharmacokinetic mechanism of the herb-drug interaction between the LDXGT formulation and sorafenib. Oral and intravenous doses of sorafenib were used, and the animals were pre-treated with different doses of the LDXGT formulation (1 and 2 g/kg, oral administration (p.o). for five consecutive days) prior to sorafenib administration (10 mg/kg, p.o.) to investigate the oral bioavailability and pharmacokinetic interaction. Rats were pre-treated with different doses of the cytochrome P450 3A4 (CYP3A4) inhibitors grapefruit juice (3, 6 and 30 mL/day, p.o., for five consecutive days) or ketoconazole (40 mg/kg, p.o., for five consecutive days) prior to sorafenib administration (10 mg/kg, p.o.) to investigate the metabolic pathway. The levels of the AST and ALT enzymes and histopathology of liver slices were used to determine the hepatoprotective function of the LDXGT formulation on sorafenib-induced hepatotoxicity in the rats administered both treatments.

## 2. Results

### 2.1. Chromatographic Analysis

The conditions used for the isocratic separation of sorafenib from rat plasma utilized an optimal mobile phase containing acetonitrile: KH_2_PO_4_ (45:55, *v*/*v*, pH 3.0). Under this condition, sorafenib was adequately separated with the highest peak symmetries, and the retention times of sorafenib and internal standard were approximately 6.2 and 2.7 min, respectively. The HPLC chromatogram of (A) blank plasma, (B) a sorafenib standard (1 µg/mL) spiked into blank plasma and (C) a real plasma sample containing sorafenib (2.84 µg/mL) collected 8 min after sorafenib administration (3 mg/kg, i.v.) shows no significant interfering signal peak. The method validation indicated that the present method had satisfactory accuracy and precision [38,39].

### 2.2. Pharmacokinetic Interactions of the Herbal Drug with Sorafenib in Rat Plasma

The pilot study of the pharmacokinetic time to concentration curve after oral administration of a single dose of sorafenib (10, 20 and 40 mg/kg, p.o.) was tested. The drug concentration versus time profile revealed the dose-dependent pharmacokinetic properties of 10–40 mg/kg sorafenib in rat plasma.

**Figure 1:**
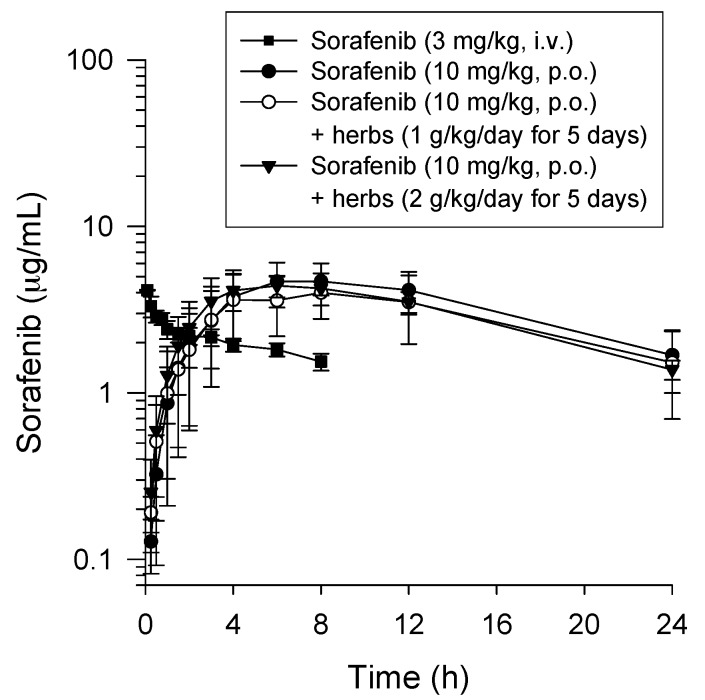
Time-concentration profiles of sorafenib in rat plasma from Group A1 (▪): sorafenib (3 mg/kg, i.v.); Group A2 (●): sorafenib (10 mg/kg, p.o.); Group A3 (○): pretreated with the Long-Dan-Xie-Gan-Tang (LDXGT) formulation (1 g/kg/day, p.o.) for five consecutive days followed by the administration of a single dose of sorafenib (10 mg/kg, p.o.); and Group A4 (▼): pretreated with the LDXGT formulation (2 g/kg/day, p.o.) for five consecutive days followed by the administration of a single dose of sorafenib (10 mg/kg, p.o.). Data are expressed as the means ± standard deviations (*n* = 6).

Representative time to concentration curves of sorafenib in rat plasma from Part A, including Groups A1–A4, are shown in Figure 1. After the intravenous administration of a single sorafenib dose (Group A1; sorafenib 3 mg/kg, i.v.), the maximal plasma concentration (Cmax) of sorafenib was rapidly detected soon after administration, followed by a slow decrease (Figure 1). Upon oral administration of a single sorafenib dose (Group A2; sorafenib 10 mg/kg, p.o.), detectable sorafenib levels were achieved in rat plasma, with maximal plasma concentrations levels (Cmax) of sorafenib observed approximately 3–8 h after administration, followed by a gradual decrease. The plasma sorafenib concentrations remained at relatively high levels after administration for four hours.

The pharmacokinetic parameters calculated from the plasma sorafenib concentrations observed in Groups A1 and A2 are summarized in Table 1. Many pharmacokinetic parameters of sorafenib, including Tmax, Cmax, AUC, half-life (t_1/2_), CL, Vss and mean residence time (MRT), were not significantly different in Groups A1 and A2. The AUCs of sorafenib in Groups A1 and A2 were 3360 ± 1906 (min·μg/mL) and 6262 ± 2259 (min·μg/mL), respectively (Table 1). The oral bioavailability (%F) of sorafenib in Group A2 was 56 ± 20% compared to Group A1 (Table 1).

### 2.3. Pharmacokinetic Interaction of the LDXGT Formulation with Sorafenib in Rat Plasma

The pharmacokinetic parameters of sorafenib, including Tmax, Cmax, AUC, t_1/2_, CL, Vss and MRT, were not significantly different among Groups A2–A4. The detectable sorafenib levels in each group reached maximal plasma concentrations in approximately 3–8 h after administration and then gradually decreased. As a result, in each group, the plasma concentrations of sorafenib excited constantly over 24 h after sorafenib administration.

The AUCs of sorafenib in Groups A2–A4 were 6262 ± 2259 (min·μg/mL) and 5653 ± 2727 (min·μg/mL) and 5489 ± 624 (min·μg/mL), respectively. Compared to Group A1, the bioavailabilities (%F) of sorafenib in Groups A3 and A4 were 50 ± 24% and 49 ± 5.0%, respectively (Table 1).

### 2.4. The Pharmacokinetics of the Effects of CYP3A4 Inhibitors (Grapefruit Juice and Ketoconazole) on Sorafenib Metabolism in Rat Plasma

**Figure 2:**
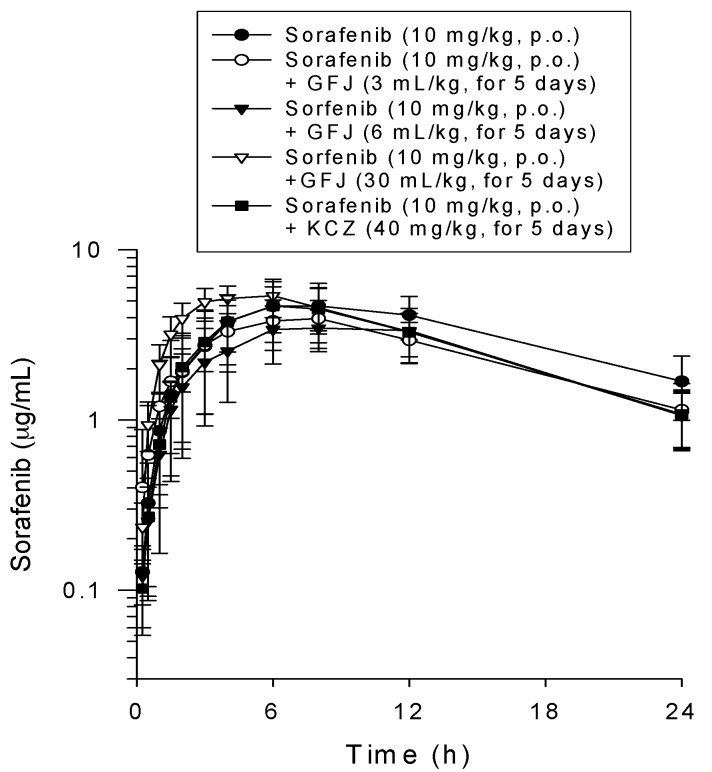
Time-concentration profiles of sorafenib in rat plasma following pretreatment with cytochrome P450 3A4 (CYP3A4) inhibitors of grapefruit juice and ketoconazole. Group A2 (●): sorafenib (10 mg/kg, p.o.); Group B1 (○): pretreated with grapefruit juice (3 mL/day, p.o.; GFJ3) for 5 consecutive days followed by the administration of a single dose of sorafenib (10 mg/kg, p.o.); Group B2 (▼): pretreated with grapefruit juice (6 mL/day, p.o.; GFJ6) for 5 consecutive days followed by the administration of a single dose of sorafenib (10 mg/kg, p.o.); Group B3 (∇): pretreated with grapefruit juice (30 mL/day, p.o.; GFJ30) for 5 consecutive days followed by the administration of a single dose of sorafenib (10 mg/kg, p.o.); and Group B4 (▪): pretreated with ketoconazole (40 mg/kg/day, p.o.; KCZ40) for 5 consecutive days followed by the administration of a single dose of sorafenib (10 mg/kg, p.o.). Data are expressed as the means ± standard deviations (*n* = 6).

Twenty-four subjects completed both treatment periods and were valid for the pharmacokinetic analysis. The pharmacokinetic time to concentration curve of the groups included in the CYP3A4 oxidative metabolism study (Groups B1–B4) are shown in Figure 2.

The pharmacokinetic parameters estimated from the plasma sorafenib concentrations following pretreatment with grapefruit juice and ketoconazole are presented in Table 1. The pharmacokinetic parameters of Groups A2, B1–B4 were compared. The pharmacokinetic parameters of sorafenib, including Tmax, Cmax, AUC, t_1/2_, CL, Vss and MRT, were not significantly different between these groups. The detectable sorafenib levels in each group achieved the maximal plasma concentrations approximately 3–8 h after administration and then gradually decreased. Furthermore, the plasma level of sorafenib was continuously maintaining at relative high since reaching the Cmax.

The AUCs of sorafenib in Groups A2, B1–B4 were 6262 ± 2259 (min·μg/mL), 5067 ± 1127 (min·μg/mL), 4399 ± 1537 (min·μg/mL), 5270 ± 1409 (min·μg/mL) and 4713 ± 1532 (min·μg/mL), respectively (Table 1). Consequently, the mean time to concentration profiles for sorafenib did not show significant differences when the groups were pretreated with the CYP3A4 inhibitors (grapefruit juice and ketoconazole) (Figure 2). Co-administration of grapefruit juice (3, 6 or 30 mL/day) or ketoconazole (400 mg/kg/day) did not affect the pharmacokinetics of the oral administration of a single sorafenib dose.

Compared to Group A1, the bioavailability (%F) of sorafenib in Groups B1–B4 was 45 ± 11%, 39 ± 14%, 52 ± 4.3% and 42 ± 14%, respectively. No significant difference in bioavailability (%F) of sorafenib was observed when rats were pretreated with CYP3A4 inhibitors (grapefruit juice and ketoconazole) compared to sorafenib (3 mg/kg, i.v.) alone (Table 1).

### 2.5. The Phase II Study of β-Glucuronidase-Mediated Sorafenib Glucuronidation in Rat Plasma

To investigate the glucuronidation of sorafenib, β-glucuronidase was added in the plasma sample collected from the Groups B4 and B5. Under the conditions described above, the ratio of the peak areas of the plasma sorafenib concentrations in the treatment with and without β-glucuronidase in Group C1 at 0, 1 and 2 h were 0.62, 0.58 and 0.60, and 0.55, 0.59 and 0.60, respectively. These data demonstrated that glucuronidation did not significantly contribute in this experiment. The ratio of the peak areas of plasma sorafenib concentrations in the presence of β-glucuronidase to the peak areas in the presence of sodium acetate in Group C2 at 0, 1, 2 h were 0.54, 0.48 and 0.54 and 0.54, 0.56 and 0.48. We did not identify significant differences in the effects of glucuronidation on sorafenib pharmacokinetics between the two groups.

### 2.6. Hepatotoxicity Studies of Rat Plasma after Treatment with Sorafenib and the LDXGT Formulation

**Figure 3:**
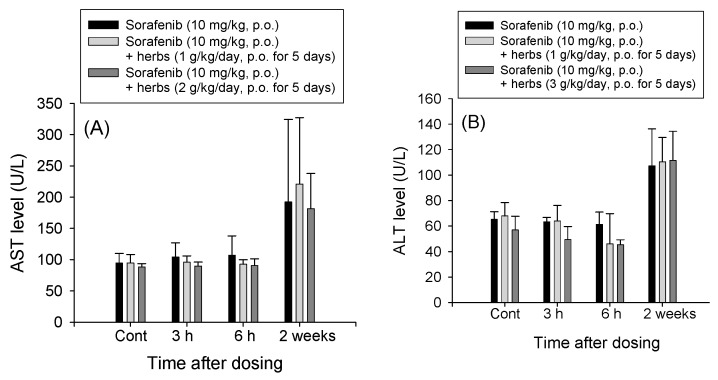
(**A**) AST and (**B**) ALT levels in rat plasma after the oral administration of sorafenib (10 mg/kg, p.o.), co-administration of LDXGT formulation (1 g/kg/day, p.o.) and sorafenib (10 mg/kg, p.o.) and co-administration LDXGT formulation (2 g/kg/day, p.o.) and sorafenib (10 mg/kg, p.o.). Blood samples were collected at 0, 3 and 6 h after the administration of the single sorafenib dose and two weeks later after the repeated administration of the dosage regimen.

Rat liver function profiles, as determined by the serum AST and ALT activities, are shown in Figure 3. No significant difference in liver function (AST and ALT) was observed among Groups D2–D4 at 0, 3 and 6 h after dosing. However, a marked elevation in liver function (AST and ALT) was observed in Groups D5–D7. When the liver functions of Groups D5–D7 were compared, no significant differences in liver function (AST and ALT) were observed between these groups (Figure 3A,B).

### 2.7. Histopathological Analyses after Treatment with Sorafenib and the LDXGT Formulation

**Figure 4:**
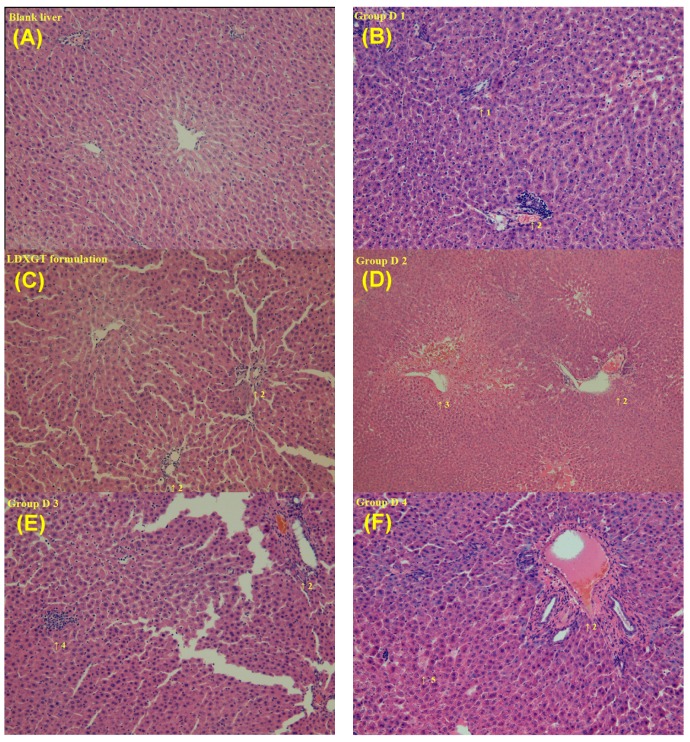
Histopathological analyses of the rat livers from groups administered a single dose of drug. (**A**) Blank liver: normal liver; (**B**) Group D1: sorafenib (3 mg/kg, i.v.); (**C**) LDXGT formulation: single dose administration of LDXGT formulation (1 g/day, p.o.); (**D**) Group D2: sorafenib (10 mg/kg, p.o.); (**E**) Group D3: pretreated with LDXGT formulation (1 g/kg/day, p.o.) for five consecutive days followed by the administration of a single dose of sorafenib (10 mg/kg, p.o.); (**F**) Group D4: pretreated with LDXGT formulation (2 g/kg/day, p.o.) for five consecutive days followed by the administration of a single dose of sorafenib (10 mg/kg, p.o.). 1: focal inflammation; 2: portal inflammation; 3: focal hemorrhage and congestion; 4: focal lytic necrosis; 5: apoptosis.

**Figure 5:**
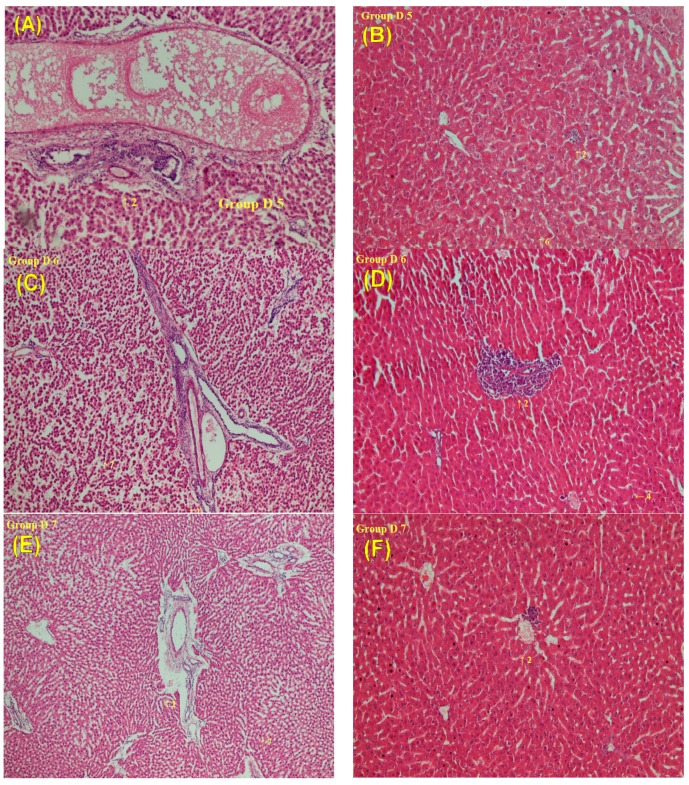
Histopathological analyses of the rat livers from groups subjected to repeated administrations. (**A**,**B**) Group D5: repeated treatment with sorafenib (10 mg/kg) for two weeks; (**C**,**D**) Group D6: repeated treatment with the combination of LDXGT formulation (1 g/kg/day, p.o.) and sorafenib (10 mg/kg, p.o.) for two weeks; (**E**,**F**) Group D7: repeated treatment with the combination of LDXGT formulation (2 g/kg/day, p.o.) and sorafenib (10 mg/kg, p.o.) for two weeks. 1: focal inflammation; 2: portal inflammation; 3: focal hemorrhage and congestion; 4: focal lytic necrosis; 5: apoptosis; 6: liver cell degeneration; 7: intercellular cohesion broken.

Histopathological analyses were performed to quantitatively evaluate the degree of liver inflammation in our experimental model using a morphometric examination of liver sections and Modified Hepatic Activity Index Grading (Modified HAI Grading; Necroinflammatory Score) [4]. The livers of control rats exhibited a normal lobular architecture, with the central vein and radiating hepatic cords in the liver slices (Figure 4A). H&E staining confirmed the presence of focal inflammation, portal inflammation, focal hemorrhage and congestion, focal lytic necrosis and hepatocyte apoptosis in the groups administered a single dose, including Groups D1–D4 (Figure 4B–F). However, in the repeated administration groups, Groups D5–D7, the liver sections revealed marked focal inflammation, portal inflammation, focal hemorrhage and congestion, focal lytic necrosis and hepatocyte apoptosis, liver cell degeneration and disrupted intercellular cohesion in the rat liver (Figure 5).

In this study, combined with liver function tests and histopathological examination, it was found that the short-term sorafenib dosing alone resulted in hepatocellular injury and inflammation, rather than hepatotoxicity. In contrast, for the long-term dosage, significant liver histopathological deterioration and hepatotoxicity were observed. Consequently, the co-administration with LDXGT would not lead to the hepatotoxicity of sorafenib.

## 3. Discussion

Despite the recent improvements in diagnostic tools and the considerable improvements in the survival rates of treated patients with HCC, the outcomes and prognoses of patients with HCC remain poor due to their poor liver function statuses and advanced cancer stages. In patients with advanced HCC, chemotherapeutic agents can provide a survival benefit. Sorafenib has become an essential tool for the management of patients with advanced HCC. However, many patients in Asia are seeking help from traditional herbal medicines due to variable degrees of chronic hepatitis and cirrhosis or the poor efficacy and severe side effects of sorafenib. Therefore, sorafenib is increasingly being administered to patients with HCC alone and in combination with Chinese herbal medicines. However, limited information about the herb-drug interaction is available. Thus, clinical studies using the sequential or simultaneous administration of combination therapies of sorafenib with Chinese herbal medicines are needed.

Because the herb-drug interactions between Chinese herbal medicines and traditional Western medicine may increase or decrease the pharmacological or toxicological effects of each formulation, a pharmacokinetic study elucidating the clinical efficacy and/or explaining and predicting a variety of events related to the efficacy and toxicity of traditional Western medicine used in combination with Chinese herbal medicines is needed [40,41]. In this context, the aim of our study was to optimize and validate an HPLC-UV experimental model to analyze the interactions of sorafenib with LDXGT, the most frequently-used Chinese herbal medicine in Taiwan, and to elucidate relations with clinical parameters (e.g., clinical outcomes and toxicity) with sensitive, selective and accurate assays.

In the clinic, patients with HCC are treated with 400–800 mg daily doses of sorafenib. The translation of this dose from human to animal studies for the present study was revisited according to the body surface area (BSA) [42]. The time to concentration curve of sorafenib after oral administration (10, 20 and 40 mg/kg, p.o.) obtained in our pilot study was tested. The time to concentration profile suggested dose-dependent pharmacokinetic properties of sorafenib within the range of 10, 20 and 40 mg/kg in rats. In the current study, we compared the pharmacokinetic parameters and bioavailability in Groups A1–A3, but did not observe significant differences between groups. Thus, no herbal-drug pharmacokinetic interactions were proven in this study. Based on these findings, the concomitant use of sorafenib and variable doses of the LDXGT formulation is safe from a pharmacokinetic perspective.

Sorafenib is frequently used in combination with one or more drugs in the clinic, rather than as a sole regimen [43,44]. Sorafenib has known drug-drug interactions with many Western medicines, such as irinotecan, docetaxel, doxorubicin, fluorouracil, etc. Sorafenib also has known pharmacokinetic interactions with CYP3A4 inducers, such as rifampin, phenytoin, carbamazepine, phenobarbital and dexamethasone [45]. According to recent clinical and preclinical studies, the LDXGT formulation and other Chinese herbal medicines exert potential hepatoprotective, immunomodulatory and antitumor effects. Therefore, the combination of sorafenib with the LDXGT formulation has been thought to exert a synergistic effect on chronic hepatitis and may even be beneficial for HCC [21,22,23,24]. However, the herb-drug interaction is limited and under-evaluated. In this study, we developed and validated a systematically-investigated assay to measure the potential herb-drug interactions of sorafenib in combination with the LDXGT formulation. When sorafenib was co-administered with variable doses of the LDXGT formulation, the plasma sorafenib concentrations did not show a significant change in pharmacokinetics. Gentiopicroside, baicalin and baicalein are three major components of LDXGT. In in vitro studies, gentiopicroside leads to a significant increase of CYP1A2, and CYP3A4 activity was observed at high concentrations; but baicalein inhibited CYP3A4 enzyme activity, and baicalin inhibits the activity of CYP1A2 in human liver microsomes [46,47,48]. Based on our result, there was no significant different between the AUC of co-administration groups and sorafenib-only group. Making a comprehensive view of LDXGT, it would not affect the CYP3A4 activity. Consequently, co-administration of variable doses of the LDXGT formulation did not affect the pharmacokinetics of sorafenib in rats.

Several research articles have concluded that sorafenib was mainly metabolized by CYP3A4 and uridine diphosphate glucuronosyl transferase 1A9 (UGT1A9) [49,50]. The pharmacokinetic parameters were estimated following treatment with the combination of sorafenib and CTY3A4 inhibitors to evaluate the effects of CYP3A4 inhibition by grapefruit juice and ketoconazole on sorafenib pharmacokinetics. The mean time to concentration profiles of sorafenib were similar between groups included in the CYP3A4 metabolism studies (Figure 2). When sorafenib-treated rats were pretreated with variable doses of grapefruit juice or ketoconazole, the pharmacokinetics of sorafenib in rats were not affected (Groups B1–B4). Thus, sorafenib was considered safe and tolerable when co-administered with both grapefruit juice and ketoconazole.

In addition to phase I oxidative metabolism, sorafenib is synchronously metabolized by the alternative phase II glucuronidation pathway. Plasma from rats pretreated with grapefruit juice (30 mL/day, p.o.; Group C1, GFJ30) or ketoconazole (40 mg/kg/day, p.o.; Group C2, KCZ40) for five consecutive days was incubated with β-glucuronidase to evaluate the effects of β-glucuronidase-mediated inhibition of phase II glucuronidation on sorafenib. The ratios of the peak area of sorafenib after β-glucuronidase incubation were similar in Groups C1 and C2. Therefore, neither the CYP3A4 inhibitors (grapefruit juice and ketoconazole), nor the glucuronidation inhibitor (β-glucuronidase) changed the pharmacokinetics of sorafenib in rats.

Previous in vitro studies of sorafenib metabolism identified two phase I prominent reactions: hydroxylation of the N-methyl group yielding metabolite M-3 and N-oxidation yielding metabolite M-2. A combination of both pathways led to the production of metabolite M-1, and demethylation of sorafenib led to the production of M-4. CYP3A4 was the enzyme responsible for phase I oxidative sorafenib metabolism. UDP-glucuronosyltransferase 1A9 (UGT1A9) was identified as the main UGT isoform catalyzing the conjugation of sorafenib with glucuronic acid to produce M-7. In contrast to humans, in vivo studies revealed that M-3 represented 12.1% and 15.6% of the AUC in rat and dog plasma, respectively. M-4 was detected in plasma from all three species. Metabolite M-2 was the main metabolite in human plasma (16.7% of AUC), but was present in small amounts in rat plasma (0.9% of AUC) and absent in dog plasma. The glucuronide of sorafenib (M-7) was a minor metabolite in human plasma and was not detected in rat or dog plasma [44]. In contrast to humans, the phase I oxidative metabolism of sorafenib observed in rats in the present study showed no significant differences following CYP3A4 inhibition and glucuronidation inhibition, possibly due to species-specific differences. However, this hypothesis requires further investigation in the future.

To date, sorafenib is the only FDA-approved target therapeutic drug for advanced HCC. Unfortunately, sorafenib has a number of adverse effects, which have led to treatment interruption and failure [50]. Among the adverse effects, hand-foot syndrome and hepatotoxicity are relatively common events that led to a discontinuation of treatment. Many investigators have attempted to combine sorafenib with other agents, including Chinese herbal medicines, to minimize the dose of sorafenib, thereby ameliorating its side effects [51]. The LDXGT formulation, a famous Chinese herbal formula, has been used to treat subjects with chronic hepatitis for thousands of years in Mainland China, Southeast Asia and Taiwan and is currently the most commonly prescribed Chinese herbal formula for subjects with chronic hepatitis in Taiwan [20]. According to a recent review, the LDXGT formulation exhibits hepatoprotective [35]), immunomodulatory [24] and anti-viral effects [36]. As shown in an in vitro investigation, the LDXGT formulation has significant cytotoxic and apoptotic effects on both HL60 and HT29 cancer cell lines [52].

In contrast, another large-scale study from the National Health Insurance (NHI) database of Taiwan concluded that prescribing the herbal medicines Xiao-Chai-Hu-Tang and Longdan Xiegan Tang to HBV-infected patients might increase their risks of liver injury [53]. Thus, the hepatoprotective or synergistic effects of the LDXGT formulation on chronic hepatitis and sorafenib-induced hepatitis in patients with HCC remain unclear. In the present study, we utilized a rat model to investigate the hepatotoxicity and histopathological changes in the rat liver following treatment with sorafenib in combination with the LDXGT formulation. Based on the results of the hepatotoxicity assay, the administration of a single sorafenib dose to the combination groups (Groups D2–D4) generated marked histopathological changes (including portal inflammation, focal hemorrhage and congestion, focal lytic necrosis, apoptosis and liver cell degeneration), but not increased AST/ALT levels.

Thus, the oral administration of a single sorafenib dose produced significant drug-induced hepatotoxicity, and co-administration of single and variable doses of the LDXGT formulation (1 g/kg/day, p.o. and 2 g/kg/day, p.o.) did not reverse the sorafenib-induced histopathological changes in the rat liver. Additionally, the co-administration of repeated doses of sorafenib and the LDXGT formulation (Groups D5–D7) revealed significant increases in the AST/ALT levels in rat plasma and marked pathological changes in the rat liver, particularly liver cell degeneration and disrupted intercellular cohesion. Thus, the repeated oral administration of sorafenib for two weeks induced marked hepatotoxicity and histopathological inflammatory changes. Repeated administration of the combination of sorafenib and the LDXGT formulation did not improve the sorafenib-induced hepatotoxicity and histopathological changes.

Hepatotoxicity is a relatively common adverse event occurring in 23–40% of patients with HCC who are treated with tyrosine kinase inhibitors (TKIs), and it is manifested by increased ALT and AST levels [54]. The histopathological alterations characteristic of TKI-induced hepatotoxicity has been described as sinusoidal congestion, hepatocyte necrosis, inflammation and hepatocyte loss around the hepatic venue in the medical literature [55,56]. Similar histopathological alterations (lobular hepatitis with mononuclear cell infiltration and hepatocyte necrosis) characteristic of sorafenib-induced hepatotoxicity have been reported in many case reports [57,58,59,60]. In our study, sorafenib-induced hepatotoxicity and histopathological alterations were observed following the administration of single and repeat doses of sorafenib to rats, as well as the administration of the combination of sorafenib and variable doses of the LDXGT formulation. Therefore, the possibility that sorafenib induces liver failure independent of previously existing liver disease should be considered, and post-treatment liver function profiles should be monitored regularly.

The LDXGT formulation is recorded in ancient Chinese classics of medicine as formulas that clear heat from the Qi level. The action of the LDXGT formulation is to drain excess heat/fire from the liver and gallbladder channels and drain the liver damp heat in the lower Jiao. In modern Chinese medicine, the LDXGT formulation serves as an agent for the treatment of jaundice, cystitis, conjunctival congestion, earache and inferior eczema of the scrotum and extremities, and the formula is reasonably used to treat chronic inflammation of the gall bladder and liver [61]. However, based on the current study, repeated treatment with the combination of variable doses of the LDXGT formulation and sorafenib did not reverse the sorafenib-induced hepatotoxicity and histopathological alterations.

The mechanism by which sorafenib induces hepatotoxicity remains unclear. A review of the literature of liver biopsies revealed centrilobular hepatocellular necrosis and lymphoplasmacellular and granulocytic infiltration of the portal tracts with significant eosinophilia, consistent with hyperallergic drug reactions. Furthermore, a subsequent lymphocyte transformation test revealed a significant increase in the stimulation index of 4.8 in subjects treated with sorafenib compared with an unspecific drug stimulation, indicating an immunoallergic drug reaction [59]. To the best of our knowledge, few review articles have documented the immunomodulatory action of the LDXGT formulation. We hypothesize that the LDXGT formulation cannot inhibit the immunoallergic drug reaction induced by sorafenib. However, further studies are needed to determine the efficacy of the LDXGT formulation at inhibiting the sorafenib-induced immunoallergic drug reaction.

## 4. Materials and Methods

### 4.1. Chemicals and Reagents

Sorafenib (BAY 43-9006; purity higher than 99% by HPLC) was kindly provided by Bayer^®^ Pharmaceutical Co., Ltd. (Kaiser-Wilhelm-Allee, Leverkusen, Germany). The *Radix Gentianae* formulation was purchased from Sun Ten Pharmaceutical Co., Ltd. (Xindian, Taiwan). The internal standard (diethylstilbestrol; purity = 99% for HPLC) was purchased from Sigma-Aldrich Chemicals (St. Louis, MO, USA). Liquid chromatography-grade acetonitrile, methanol and potassium dihydrogen phosphate monohydrate (KH_2_PO_4_·H_2_O) were purchased from E. Merck (Darmstadt, Germany). Triply deionized water (Millipore, Bedford, MA, USA) was used for all aqueous solutions. All of the other reagents were of analytical grade.

### 4.2. Sample Preparation

Sorafenib and internal standard stock solutions were prepared by precisely weighing the respective compounds and dissolving them in 100% methanol and subsequently diluting them with 50% methanol to the indicated concentrations. Calibration standards and internal standard solutions were stored at −20 °C for drug administration in the animal experiment. The LDXGT formulation was dissolved in triply deionized water and prepared for drug administration in the animal experiment.

### 4.3. Animals

Male Sprague-Dawley rats (National Yang-Ming University Animal Center, Taipei, Taiwan) were housed in different cages on a 12-h light/dark cycle. Free access to food (Laboratory Rodent Diet 5001, PMI Feeds, Richmond, IN, USA) and water was provided at all times. Animal experimental protocols were reviewed and approved by the Institutional Animal Experimentation Committee of the National Yang-Ming University. The rats (240–300 g) were anesthetized with a dose of urethane (1 g/kg, i.p.) and remained anesthetized as needed throughout the experimental period. The rat body temperature was maintained with a heating pad.

Rats were anesthetized with urethane (1 g/kg, i.p.). A longitudinal skin incision was made over the area where the right external jugular vein passed dorsal to the pectoralis major muscle. The catheter, filled with 200 units/mL heparinized physiologic saline, was placed in the tight jugular vein and then advanced into the sinus venous. The catheter was inserted up to the first silicone stopper and anchored in place by suturing the stopper to the muscle. The free end of the catheter was passed under the skin of the dorsum of the neck just caudal to the ears and attached to the skin. Finally, the catheter was filled with 200 units/mL heparinized saline, and a plug was inserted in the free end of the catheter [62,63].

### 4.4. Sample Preparation

A 50-μL aliquot of plasma was added to 150 μL of the internal standard solution to precipitate the proteins. After vortexing, mixing and sonication for 10 seconds, all samples were centrifuged at 13,000× *g* for 10 min. The supernatant was transferred into the HPLC systems for analysis.

### 4.5. HPLC Analysis

HPLC was performed using a chromatographic pump (Model LC-20AT), an autosampler (Model SIL-20AC) and a diode array detector (Model SPD-M20A, Shimadzu, Kyoto, Japan). Chromatographic separation was performed on a C18 column (50 mm × 2.1 mm i.d.; particle size 1.7 μm, Waters Acquity, Dublin, Ireland), protected by a guard column. The mobile phase consisted of acetonitrile: KH_2_PO_4_ (45:55, *v*/*v*, pH 3.0), and the flow rate was 0.2 mL/min. Eluent A consisted of 100% acetonitrile, and Eluent B was KH_2_PO_4_ (pH 3.0). In an 80-min total running time, the linear gradient started at 5% Eluent A and 95% Eluent B to 95% Eluent A and 5% Eluent B. The pump pressure was limited to 8535 psi. The temperature of the autosampler was maintained at 10 °C. The temperature of the column was maintained at 25 °C. The UV wavelength was set at 265 nm for peak integration, and the sample injection volume was 5 μL.

For determination of gentiopicroside, baicalin and baicalein in the LDXGT pharmaceutical products, the quantitation process is as follows. The powder of LDXGT pharmaceutical products were weight as 5 g and suspended in 5 mL water, then solicited in the ultrasonic shock machine for 10 min. After that, the supernatant was collected and analyzed by HPLC. The determination means of components were achieved by HPLC performed using Shimadzu HPLC (LC-20AT, Kyoto, Japan). Shimadzu HPLC equipment was composed of a diode array detector (Model SPD-M20A, Shimadzu, Kyoto, Japan), an LC-10AT pump and an SIL-20AC automatic injector. Three analytes, gentiopicroside, baicalin and baicalein, were separated on a C18 column (250 mm × 4.6 mm i.d.; particle size 5 μm, Synergi™ Fusion-RP column (Phenomenex^®^, Macclesfield, UK) with the mobile phase consisting of acetonitrile (containing 0.1% formic acid, Eluent A) and NH_4_HCO_3_ (adjusted by H_3_PO_4_, pH 7.3, Eluent B). The gradient mobile phase system was performed as starting at 20% Eluent A, then maintaining for 3 min. The linear gradient was shifted from 20% to 55% Eluent A for the next 7 min. After that, the percentage of Eluent A was increased to 90% within another 10 min. The flow rate was 1.0 mL/min; the UV wavelength was set at 273 nm; and the sample injection volume was 10 μL. The results demonstrated that the contents of the four components of LDXGT were quantitated as follows (mg/g): gentiopicroside 17.62 mg/g, baicalin 10.58 mg/g and baicalein 0.19 mg/g in LDXGT.

### 4.6. Experimental Study Design

The LDXGT formulation products used in this investigation were purchased from Sun Ten Pharmaceutical Co., Ltd., and their batch number was 031342. The LDXGT formulation consists of Radix Gentianae (4.0 g), (2.0 g), Fructus Gardenia (2.0 g), Rhizoma Alismatis (4.0 g), Caulis Akebiae (2.0 g), Semam Plantaginis (2.0 g), Radix Angelicae sinensis (2.0 g), Radix Rehmanniae (2.0 g), Radix Bupleuri (4.0 g) and Radix Glycyrrhizae (2.0 g). All herbs were extracted, and then yielded an amount of LDXGT dry extract, which was a 5.2-time extraction of the original LDXGT decoction. Finally, the ultimate extraction, cornstarch and powder cellulose were mixed and granulated with their ratio being 10:7:7. The LDXGT formulation granulated powder was administered, which was suspended in water. Then, rats were given with the LDXGT suspension solution. The whole experiment was divided into the following four main parts to the mechanisms by which the herb-drug interaction between the LDXGT formulation and sorafenib affected hepatotoxicity, histopathology and pharmacokinetics: (A) herbal drug pharmacokinetic interaction; (B) CYP3A4 metabolism; (C) phase II glucuronidation and (D) hepatotoxicity and histopathological studies.

#### 4.6.1. Part A: Herbal Drug Pharmacokinetic Interaction Study

- Group A1, sorafenib (3 mg/kg, i.v.).
- Group A2, sorafenib (10 mg/kg, p.o.).
- Group A3, pretreated with the LDXGT formulation (1 g/kg/day, p.o.) for 5 consecutive days followed by the administration of single dose of sorafenib (10 mg/kg, p.o.).
- Group A4, pretreated with the LDXGT formulation (2 g/kg/day, p.o.) for 5 consecutive days followed by the administration of a single dose of sorafenib (10 mg/kg, p.o.).

#### 4.6.2. Part B: CYP3A4 Metabolism Study

- Group B1, pretreated with grapefruit juice (3 mL/day, p.o.; GFJ3) for 5 consecutive days followed by the administration of a single dose of sorafenib (10 mg/kg, p.o.).
- Group B2, pretreated with grapefruit juice (6 mL/day, p.o.; GFJ6) for 5 consecutive days followed by the administration of a single dose of sorafenib (10 mg/kg, p.o.).
- Group B3, pretreated with grapefruit juice (30 mL/day, p.o.; GFJ30) for 5 consecutive days followed by the administration of a single dose of sorafenib (10 mg/kg, p.o.).
- Group B4, pretreated with ketoconazole (40 mg/kg/day, p.o.; KCZ40) for 5 consecutive days followed by the administration of a single dose of sorafenib (10 mg/kg, p.o.).

#### 4.6.3. Part C: Phase II Glucuronidation Study

- Group C1, pretreated with grapefruit juice (30 mL/day, p.o.; GFJ30) for 5 consecutive days followed by the administration of a single dose of sorafenib (10 mg/kg, p.o.).
- Group C2, pretreated with ketoconazole (40 mg/kg/day, p.o.; KCZ40) for 5 consecutive days followed by the administration of a single dose of sorafenib (10 mg/kg, p.o.).

#### 4.6.4. Part D: Hepatotoxicity and Histopathological Studies

- Group D1, sorafenib (3 mg/kg, i.v.).
- Group D2, sorafenib (10 mg/kg, p.o.).
- Group D3, pretreated with the LDXGT formulation (1 g/kg/day, p.o.) for 5 consecutive days followed by the administration of a single dose of sorafenib (10 mg/kg, p.o.).
- Group D4, pretreated with the LDXGT formulation (2 g/kg/day, p.o.) for 5 consecutive days followed by the administration of a single dose of sorafenib (10 mg/kg, p.o.).
- Group D5, repeated treatment with sorafenib (10 mg/kg) for 2 weeks.
- Group D6, repeated treatment with the combination of the LDXGT formulation (1 g/kg/day, p.o.) and sorafenib (10 mg/kg, p.o.) for 2 weeks.
- Group D7, repeated treatment with the combination of the LDXGT formulation (2 g/kg/day, p.o.) and sorafenib (10 mg/kg, p.o.) for 2 weeks.

### 4.7. The Herbal Drug Pharmacokinetic Interaction Study of Sorafenib (Part A)

The jugular-catheterized rats were randomly divided into four groups, Groups A1–A4, as described above. An aqueous solution of the LDXGT formulation was administered to rats by oral gavage at doses of 1 g/kg and 2 g/kg. The aqueous sorafenib solution was administered to rats by oral gavage at a dose of 10 mg/kg. After administration, blood samples were collected (0.15 mL) from the rats’ jugular veins at the following time intervals: 0.5, 1, 1.5, 2, 3, 4, 6, 8, 12 and 24 h after drug administration. The blood samples were centrifuged at 13,000× *g* for 10 min to obtain the plasma and stored at −20 °C until the HPLC analysis. Data from these samples were used to construct pharmacokinetic profiles by plotting drug concentrations versus times.

### 4.8. Study of Phase I CYP3A4-Mediated Sorafenib Metabolism (Part B)

Rats were divided into Groups B1–B4 containing six animals each as described above to evaluate the effects of CYP3A4 inhibitors (grapefruit juice and ketoconazole) on sorafenib pharmacokinetics. An aqueous grapefruit juice solution was administered to rats by oral gavage at doses of 3 mL/day, 6 mL/day and 30 mL/day. The aqueous ketoconazole solution was administered to rats by oral gavage at a dose of 40 mg/kg/day. The aqueous sorafenib solution was orally administered to rats at a dose of 10 mg/kg/day. Blood samples (0.15 mL) were collected from the jugular vein at the following time intervals after drug administration: 0.25, 0.5, 1, 2, 4, 8, 12 and 24 h. The blood samples were centrifuged at 13,000× *g* for 10 min, and the resulting plasma samples were stored at −20 °C until the HPLC analysis.

### 4.9. Study of the Phase II Glucuronidation Metabolism of Sorafenib (Part C)

Plasma samples were collected from the groups pretreated with grapefruit juice (30 mL/day, p.o.; GFJ30) and pretreated with ketoconazole (40 mg/kg/day, p.o.; KCZ40) for 5 consecutive days followed by the administration of a single dose of sorafenib (10 mg/kg, p.o.). The plasma sample was incubated with β-glucuronidase (diluted to 1 unit/μL in buffer) in a reaction mixture containing 100 mM sodium acetate buffer (pH 5.0) in a final volume of 100 μL. Following the incubation, the plasma (100 μL) was then mixed with 60 μL of MeOH containing the internal standard for protein precipitation. Before initiating the glucuronidation reactions, all the samples were placed in a 37 °C water bath and incubated for 0, 1 and 2 h. The denatured protein precipitate was separated by vortexing for 5 min and centrifuging at 13,000× *g* for 10 min. The supernatant was filtered through a 0.22 µm mini-filter and analyzed using the HPLC-UV system.

### 4.10. Hepatotoxicity and Histopathological Studies of Sorafenib (Part D)

Hepatotoxicity and histopathological experiments were performed on 42 jugular-catheterized rats that were randomly divided into Groups D1–D7 containing six animals, as described above. In the “Part D” study, an aqueous solution of the LDXGT formulation and sorafenib were each administered to rats by oral gavage. Blood samples were collected from the jugular vein at 0, 0.5 and 1 h after dosing for the intravenous administration group. For each group treated with a single dose (Groups D1–D4), blood samples (0.15 mL) were collected from the jugular veins at 0, 3 and 6 h after dosing. For each group administered repeated doses (Groups D5–D7), blood samples (0.15 mL) were collected from the jugular veins at 2 weeks after dosing. Serum was obtained by centrifugation. The levels of the AST and ALT enzymes were determined using an automatic biochemical analysis. After blood sampling, the livers were dissected from the rats in each group and prepared using a series of processes. Pathological observations of liver slices were used to confirm the histopathological alterations in the liver tissues induced by single and repeated doses of sorafenib following pretreatment with the LDXGT formulation, which was conducted by a pathologist from the Department of Pathology of Taipei City Hospital Ren-Ai branch (Taipei, Taiwan).

### 4.11. Pharmacokinetic Analysis

All data were subsequently processed using the WinNonlin Version 5.0 program (Pharsight Corporation, Mountain View, CA, USA). The following pharmacokinetic parameters were calculated: half-life (t_1/2_; min), peak concentration (C*_max_*; μg/mL), area under the concentration versus time curve (AUC; min μg/mL), clearance (CI; mL/min/kg), apparent volume of distribution at steady state (Vss; mL/kg) and mean residence time (MRT; min). The area under the plasma time to concentration curve from zero to infinity (AUC_0−∞_) was calculated using the trapezoidal rule, with extrapolation to infinity for the terminal elimination rate constant (Ke) for sorafenib. The significance of the differences was assessed using Student’s *t*-test. Experimental data and the pharmacokinetic parameters were expressed as the means ± standard deviation.

## 5. Conclusions

In this study, an accurate and validated HPLC-UV method was developed to determine the sorafenib concentrations in rat plasma and applied to the studies of sorafenib pharmacokinetics and metabolism. We did not identify potential herb-drug interactions of sorafenib with the LDXGT formulation in the pharmacokinetic study. Furthermore, co-administration of variable, single and repeated doses of the LDXGT formulation did not suppress or exacerbate the hepatotoxicity and histopathological alterations induced by sorafenib in rats. Thus, we conclude that the combination of sorafenib and the LDXGT formulation is safe, but does not ameliorate sorafenib-induced hepatotoxicity.

## Figures and Tables

**Table 1:** Pharmacokinetic parameters in rats treated with sorafenib alone and pretreated with the LDXGT formulation, grapefruit juice and ketoconazole.

	Parameters	AUC (min·μg/mL)	T_max_ (min)	C_max_ (μg/mL)	t_1/2_ (min)	CL (mL/min/kg)	V_ss_ (mL/kg)	F (%)
Groups								
Group A1		3360 ±1906	-	4.6 ± 0.2	499 ± 275	1.1 ± 0.4	1189 ± 129	
Group A2		6262 ± 2259	420 ± 66	5.0 ± 1.4	629 ± 118	1.8 ± 0.7	1564 ± 477	56 ± 20
Group A3		5653 ± 2727	380 ± 192	4.8 ± 1.0	649 ± 192	2.1 ± 0.8	1801 ± 497	50 ± 24
Group A4		5489 ± 624	310 ± 110	4.8 ± 0.7	594 ± 153	1.8 ± 0.2	1565 ± 231	49 ± 5.0
Group B1		5067 ± 1127	400 ± 62	4.3 ± 0.9	577 ± 153	2.0 ± 0.4	1689 ± 550	45 ± 11
Group B2		4399 ± 1537	480 ± 131	3.9 ± 1.2	545 ± 108	2.6 ± 1.1	1950 ± 678	39 ± 14
Group B3		5270 ± 1409	336 ± 54	5.5 ± 1.1	457 ± 49	2.1 ± 0.8	1345 ± 469	52 ± 4.3
Group B4		4713 ± 1532	440 ± 62	4.9 ± 1.9	435 ± 50	2.3 ± 0.8	1443 ± 437	42 ± 14

AUC: area under the time to concentration curve; T_max_: time of maximum concentration; C_max_: maximum concentration; t_1/2_: half-life; CL: clearance; V_ss_: apparent volume of distribution at steady state; F: oral bioavailability; Group A1: sorafenib (3 mg/kg, i.v.); Group A2: sorafenib (10 mg/kg, p.o.); Group A3: pretreated with LDXGT formulation (1 g/kg/day, p.o.) for 5 consecutive days followed by the administration of a single dose of sorafenib (10 mg/kg, p.o.); Group A4: pretreated with LDXGT formulation (2 g/kg/day, p.o.) for 5 consecutive days followed by the administration of a single dose of sorafenib (10 mg/kg, p.o.); Group B1 (GFJ3): pretreated with grapefruit juice (3 mL/day, p.o.; GFJ3) for 5 consecutive days followed by the administration of a single dose of sorafenib (10 mg/kg, p.o.); Group B2 (GFJ6): pretreated with grapefruit juice (6 mL/day, p.o.; GFJ6) for 5 consecutive days following single dose of sorafenib (10 mg/kg, p.o.); Group B3: pretreated with grapefruit juice (30 mL/day, p.o.; GFJ30) for 5 consecutive days followed by the administration of a single dose of sorafenib (10 mg/kg, p.o.); Group B4: pretreated with ketoconazole (40 mg/kg/day, p.o.; KCZ40) for 5 consecutive days followed by the administration of a single dose of sorafenib (10 mg/kg, p.o.). Data are expressed as the means ± standard deviations; means were obtained from six rats in each group.
